# A new biomarker to examine the role of hippocampal function in the development of spatial reorientation in children: a review

**DOI:** 10.3389/fpsyg.2015.00490

**Published:** 2015-04-24

**Authors:** Vanessa Vieites, Alina Nazareth, Bethany C. Reeb-Sutherland, Shannon M. Pruden

**Affiliations:** Department of Psychology, Florida International University, Miami, FL, USA

**Keywords:** eyeblink conditioning, hippocampus, spatial memory, spatial navigation, spatial reorientation

## Abstract

Spatial navigation is an adaptive skill that involves determining the route to a particular goal or location, and then traveling that path. A major component of spatial navigation is spatial reorientation, or the ability to reestablish a sense of direction after being disoriented. The hippocampus is known to be critical for navigating, and has more recently been implicated in reorienting in adults, but relatively little is known about the development of the hippocampus in relation to these large-scale spatial abilities in children. It has been established that, compared to school-aged children, preschool children tend to perform poorly on certain spatial reorientation tasks, suggesting that their hippocampi may not be mature enough to process the demands of such a task. Currently, common techniques used to examine underlying brain activity, such as electroencephalography (EEG) and functional magnetic resonance imaging (fMRI), are not suitable for examining hippocampal development in young children. In the present paper, we argue instead for the use of eyeblink conditioning (EBC), a relatively under-utilized, inexpensive, and safe method that is easy to implement in developing populations. In addition, EBC has a well defined neural circuitry, which includes the hippocampus, making it an ideal tool to indirectly measure hippocampal functioning in young children. In this review, we will evaluate the literature on EBC and its relation to hippocampal development, and discuss the possibility of using EBC as an objective measure of associative learning in relation to large-scale spatial skills. We support the use of EBC as a way to indirectly access hippocampal function in typical and atypical populations in order to characterize the neural substrates associated with the development of spatial reorientation abilities in early childhood. As such, EBC is a potential, simple biomarker for success in tasks that require the hippocampus, including spatial reorientation.

## Introduction

Imagine being unable to mentally retrace your steps in order to travel back home, or being unable to retain any memory for the locations of household objects. Remembering where you left your keys, finding your car in a crowded parking lot, and navigating through an unfamiliar neighborhood are all tasks that require the use of spatial memory, the kind of memory that allows us to recall information about our three-dimensional environment, such as the positions of objects relative to other environmental features. Spatial memory functions include spatial navigation, which is the ability to determine a route and then travel that route to a specific location, and its related component, spatial reorientation, which is the ability to reestablish a sense of position after being lost in the environment. These skills, known collectively as large-scale spatial abilities, are adaptive functions that are crucial to the survival of humans and other animals (Newcombe et al., 2013). As adults, we often take these skills for granted; however, achieving expertise in these spatial tasks takes time to develop through the interaction between brain development and individual experience.

One area of the brain found to be vital for spatial navigation is the hippocampus (e.g., O’Keefe and Nadel, 1978; Morris et al., 1982; Abrahams et al., 1999; Burgess et al., 2002; Wills et al., 2014). The specific role of the hippocampus in spatial reorientation is less conclusive, but the hippocampus has been implicated in certain processes for reorientation (for a review, see Sutton and Newcombe, 2014). While much is understood about the role of the hippocampus in various components of adult spatial memory (for a review, see Burgess et al., 2002), relatively little is known about the development of the hippocampus in relation to children’s spatial capacities, specifically spatial reorientation abilities.

Contributing to our lack of knowledge of hippocampal development in relation to spatial development is the lack of age-appropriate methods that may be used to examine hippocampal functioning. Popular techniques, such as electroencephalography (EEG) and functional magnetic resonance imaging (fMRI), typically used to explore brain function, are not ideal for examining hippocampal development in young children. Specifically, while EEG is a suitable technique to examine infant and child neural activity, its low spatial resolution does not allow one to examine specific subcortical regions such as the hippocampus. In contrast, while *f*MRI has superior spatial resolution and has been successfully used to examine hippocampal functioning in relation to spatial navigation (Rodriguez, 2010) and reorientation (Sutton et al., 2010, 2012) in adults (although see Devlin et al., 2000 and Veltman et al., 2000, for discussions on susceptibility artifacts), it is near impossible to use this technique with children younger than 5 years of age while they are awake due to the amount of movement artifact that is present in this population. Here we suggest using an alternative technique, Pavlovian eyeblink conditioning (EBC), to indirectly measure hippocampal functioning in young children in order to further our understanding of the complex interaction between hippocampal development and spatial reorientation performance. EBC has a well-defined neural circuitry, which includes the hippocampus, is non-invasive, and can be employed in typical and atypical pediatric populations, making it a potential tool that can be used to investigate hippocampal development and its relation to spatial reorientation abilities in young children.

In the current review, we will provide an overview of research on spatial cognition in relation to the hippocampus, but specifically focus on the development of spatial reorientation in children and the candidate neural mechanisms involved in such development. We choose to focus on spatial reorientation because it is a popular, quick, and practical measure of large-scale spatial ability (i.e., non-paper-and-pencil task) used with children in a real-world, three-dimensional space. Furthermore, we will discuss the benefits of using EBC as a non-invasive tool to indirectly examine the development of hippocampal function, and focus on the potential use of this measure as a proxy for hippocampal involvement in the development of spatial reorientation abilities in typical and atypical populations. We propose that if hippocampal development indeed underlies developmental differences observed in spatial reorientation tasks during early childhood, then similar differences should be observed in hippocampal-dependent EBC tasks. Moreover, individual performance on one task should potentially be associated with performance on the other task.

## Behavioral Development of Spatial Reorientation

Spatial reorientation relies on the broader cognitive domain of spatial memory, which involves the ability to mentally represent objects, other visual features, and their relations to each other within the environment. This type of memory allows humans and other animals to keep track of their bodies’ position, to know in which direction they are headed, and to search for hidden objects and/or food (Newcombe et al., 2013). There are two potent types of environmental cues used to reorient oneself in the appropriate direction: (1) geometric cues (e.g., length, distance, angles) and (2) featural or landmark cues (e.g., trees, buildings, street signs; Lyons et al., 2014). Adults frequently combine geometric and featural cues to guide their spatial navigation and reorientation (Ratliff and Newcombe, 2008), whereas young children show a preference for geometric cues (Hermer and Spelke, 1996; Lee and Spelke, 2010). Specifically, in studies where children are asked to find the location of an object that is hidden in a corner of a rectangular enclosure near a featural cue (i.e., colored wall) after being disoriented, children under the age of 6 years will typically search for the object in either the correct corner or the rotationally equivalent, opposite corner (Hermer and Spelke, 1994, 1996). Thus, they are employing the geometric shape of the enclosure to determine the location of the object, and disregarding the landmark (i.e., colored wall), which is a better indicator than geometry of the object’s location in this particular environment. In contrast, children 6 years of age and older typically use the featural cue to find the hidden object (Hermer-Vazquez et al., 2001). This dissociation between the use of geometric and featural cues in the development of spatial reorientation in young children has been the focus of many debates, and has led to the development of several theories over the past 25 years (e.g., Cheng, 1986; Newcombe and Huttenlocher, 2006; Miller and Shettleworth, 2008; Stürzl et al., 2008). For a review of these theories and subsequent debates, see Cheng et al. (2013).

While it is typical to observe that children younger than 6 years of age use geometric rather than featural or landmark cues when reorienting in the appropriate direction, this phenomenon can be influenced by various experiential factors (e.g., Twyman and Newcombe, 2010). For example, spatial reorientation abilities are malleable across development, meaning that the dependence on geometric or featural cues to reorient not only changes across early childhood (Twyman et al., 2013), but is also affected by factors such as language use (e.g., Hermer-Vazquez et al., 1999; Shusterman et al., 2011) and experience with reliable cues (Ratliff and Newcombe, 2008).

Specifically, regarding language use, it has been suggested that the emergence of spatial language, particularly directional terms (e.g., left, right, above, below), accounts for the ability of 6-year-olds, but not younger children, to use landmark features to locate hidden objects after being disoriented in small enclosures (e.g., Hermer-Vazquez et al., 2001; Shusterman et al., 2011). Improved spatial reorientation performance among 4-year-old children after being taught left and right directions (Shusterman, 2006) supports the idea that language experience significantly influences spatial reorientation. In addition to language experience, previous exposure to the environment, as well as the size of the space, boosts spatial reorientation abilities in children younger than 6 years of age. For example, 3-year-old children can use landmark cues if they are allowed to explore the room before being tested on the spatial reorientation task (Learmonth et al., 2008), suggesting that observing the relevant featural information needed to locate the object is sufficient to elicit success on the task. In addition, toddlers and children under the age of 6 years are more likely to use featural cues when tested in a large (e.g., 8′ × 12′) rather than small (e.g., 4′ × 6′) enclosure (Learmonth et al., 2001, 2002). Room size similarly affects spatial reorientation in adults (Ratliff and Newcombe, 2008) as well as animals (Sovrano et al., 2005; Chiandetti and Vallortigara, 2008).

It has been suggested that organisms rely more on geometric properties than landmarks to reorient in small spaces because it is easier to gauge, or scan, the geometric layout of small environments compared to large ones (Sovrano and Vallortigara, 2006). Therefore, featural cues within a small environment appear proximal while, in contrast, features in large rooms appear more distal. This distinction between proximal and distal cues is important because animals have shown a preference for using distal rather than proximal cues when reorienting (Hebb, 1938; O’Keefe and Nadel, 1978; Zugaro et al., 2004; Nadel and Hupbach, 2006), thus providing some explanation as to why featural or landmark cues are not typically used to reorient in small spaces. However, this does not fully explain why young children continue to perseverate on geometric cues when reorienting compared to older children. It is likely that these developmental differences at the level of behavior are linked to developmental differences at the level of the brain. Specifically, when neural structure and function related to spatial behaviors are activated, we should expect to see corresponding shifts in such behaviors. In the next section, we will focus on one particular region of the brain known to be involved in spatial memory, namely, the hippocampus.

## Neurobiology of Spatial Memory

The hippocampus of humans and other animals has long been implicated in spatial navigation (for reviews, see Burgess et al., 2002; Wills et al., 2014), and, more recently, in virtual reorientation in human adults (Sutton et al., 2010, 2012). It has been proposed that the hippocampus forms a cognitive map of the environment, representing locations and their contents, which allows us to encode spatial relations and traverse our surroundings using allocentric and egocentric frames of reference (O’Keefe and Nadel, 1978). While an egocentric strategy for navigating relies on a series of body motions and left–right turns used to arrive at the goal, an allocentric strategy relies on information outside of the body. Thus, an egocentric frame of reference is used to compute spatial relations between oneself and the goal (Zaehle et al., 2007), while an allocentric frame of reference requires one to focus on fixed environmental landmarks, locations of objects, and their relations to each other (Bohbot et al., 2004). Allocentric and egocentric frames of reference are also utilized in the learning of locations in the environment, known as place learning (Waller et al., 2000). In particular, the use of allocentric spatial strategies (i.e., mapping the relations between multiple landmarks in the environment in order to navigate) has shown to be hippocampal-dependent (Bohbot et al., 2004). Specifically, *f*MRI data revealed that the hippocampus was significantly activated in healthy participants who used spatial strategies to locate an object on a virtual 8-arm radial maze with distant landmarks; however, patients with medial temporal lobe resections, including the hippocampus, showed impaired performance (Bohbot et al., 2004).

Landmarks often serve as reliable sources of locations for animals that must constantly travel far from their home (e.g., nest) in search for food and/or water. When navigating, animals appear to rely more on landmarks that are farther away from them, also known as distal cues (for a review, see Chiandetti and Vallortigara, 2008). Previous studies have demonstrated that hippocampal lesions affect rats’ performance on place navigation (i.e., learning the location of a hidden goal after a delay period between training and testing; e.g., Morris et al., 1982; Jacobson et al., 2012). In these studies, rats with exclusive hippocampal lesions had significant impairments using allocentric spatial processing (mapping the relations among multiple, distal landmarks) to locate a hidden platform compared to control rats, which underwent either (1) superficial cortical lesions (hole drilled in other brain regions leading up to, but not including, the hippocampus), (2) sham surgery (burr holes drilled in skull, but no brain damage), or (3) no surgery. In contrast, using proximal or local cues to locate a goal has *not* been found to rely on an intact hippocampus (Pearce et al., 1998; Stackman and Herbert, 2002). In sum, these studies suggest that the processing of distal, but not proximal, landmarks in order to navigate and reorient is especially hippocampal-dependent.

The preference for using distal over proximal cues to navigate and orient oneself may be due to the firing of two kinds of cells that are dependent on the information provided by distal cues (Knierim and Rao, 2003; Zugaro et al., 2004). These neurons, place cells and head-direction cells, work together to aid in navigation and orientation, playing a key role in the brain’s spatial representation system (Moser et al., 2008). Hippocampal place cells fire when an animal occupies specific locations in the environment, known as place fields, while head-direction cells, located in various brain regions separate from the hippocampus (e.g., anterior dorsal thalamic nucleus, post-subiculum, dorsal striatum; Taube, 1998), discharge when the animal orients its head in a specific, “preferred” direction (Zugaro et al., 2001; Kubie and Fenton, 2009). Both types of cells are influenced by relatively distant landmark cues. For instance, place cells and head-direction cells have been found to fire with respect to the rotations of distal cues, as opposed to proximal cues (i.e., cues that are relatively close in proximity to the target location and/or the animal). In contrast, when distal cues remain still while proximal cues are in motion, these cells have been found *not* to shift, or rotate, along with the moving proximal cues, so they remain fixed on the unchanging landmarks (O’Keefe and Conway, 1978; Zugaro et al., 2001; Knierim and Rao, 2003; Yoganarasimha et al., 2006). In addition, when rats are exposed to proximal and distal cues rotating in conflicting directions, their place cells and head-direction cells rotate to the same degree as the distal cues instead of the proximal cues (Knierim and Rao, 2003; Zugaro et al., 2004; Yoganarasimha et al., 2006). These findings are suspected to occur because distal cues that designate meaningful locations tend to appear large and relatively motionless, making them more salient and reliable to a navigating animal.

The human hippocampus and adjacent medial temporal lobe structures have also been implicated in object-location binding (Crane and Milner, 2005), while the parahippocampus has been shown to be involved in the processing of spatial scenes (for a review, see Burgess et al., 2002; Ekstrom et al., 2003). Making object-location or object-place associations involves the use of associative memory, the formation of which is supported by the hippocampus (e.g., Goodrich-Hunsaker et al., 2009). A typical object-location task involves showing participants an array of objects on a tabletop, asking them to remember where the objects are located with respect to one another, and then asking them to replace those same objects on a blank tabletop. Aside from not being able to learn a new route (for review, see Burgess et al., 2002), patients with damage to their right hippocampus or right medial temporal lobe, in particular, show deficits in object-location tasks that (Crane and Milner, 2005), especially after their viewpoint has been shifted (Shrager et al., 2007). Furthermore, various neuroimaging studies found increased right hippocampal activation during, not only route learning, but object-location memory tasks as well (for reviews, see Burgess et al., 2002; Baumann and Mattingley, 2014). Thus, given the significance of the hippocampus in remembering where things are, and that spatial reorientation tasks involve recalling the specific location of an invisible object and linking it to a salient landmark (Lee et al., 2006), it is quite possible that the hippocampus, particularly the right hippocampus, is involved in reorienting behaviors as well.

Research on the neural basis of spatial navigation and reorientation has shed light on different underlying means of encoding spatial information (for review, see Sutton and Newcombe, 2014). O’Keefe and Nadel (1978) proposed that we use locale (i.e., allocentric, map-based representation of the environment) and taxon (i.e., egocentric, route based representation of the environment) systems for navigating. The former system relies on the hippocampus and its place cells (O’Keefe, 1976), while the latter system depends more so on the dorsal striatum (White and McDonald, 2002). Similarly, the usage of geometric and featural cues implicates a two-factor hippocampal-striatal system (For a review, see Cheng et al., 2013). Evidence from fMRI studies supports the hypothesis that the hippocampus encodes geometric information, while the striatum encodes landmark information (Doeller and Burgess, 2008; Doeller et al., 2008). For example, it was found that encoding an object’s location relative to a boundary (geometric cue) activated the hippocampus, while doing so relative to a landmark (featural cue) activated the dorsal striatum. However, in the studies conducted by Doeller et al. (2008), participants were not disoriented, so it would be difficult to draw conclusions regarding brain regions involved in reorientation per se (Sutton et al., 2010). Yet, in virtual reorientation studies, significantly greater hippocampal activation following disorientation was found in conditions where a non-geometric feature (i.e., red wall) was present (Sutton et al., 2010, 2012). The disparity in these findings implies the use of different neuropsychological mechanisms in reorienting. As such, virtual reorientation studies highlight the specific importance of the hippocampus in spatial reorientation. In sum, it appears that the hippocampus plays a significant role in remembering the locations of objects *and* binding said locations to allocentric representations of the environment. Moreover, as highlighted in the next section, typical and atypical structural changes in the hippocampus across childhood lead to functional changes such as developmental differences in memory and, potentially, spatial reorientation abilities.

## Typical and Atypical Hippocampal Development

It is now known that the hippocampus of non-human animals and humans is not fully formed at birth, but continues to develop and mature. Evidence suggests that the rat hippocampus reaches maturity between the 4^th^ (Altman and Das, 1965) and 7th (Pokorny and Yamamoto, 1981) week after birth. More recent findings show that normal human hippocampal development may continue into early adulthood (Gogtay et al., 2006). Therefore, this immaturity should be reflected in hippocampal-dependent behaviors, including spatial reorientation behaviors. Indeed, we have seen in studies on mice that compared to 5- and 9-week-old mice, 3-week-old mice had significantly more trouble locating food by orienting to distal cues on an 8 arm radial maze (Chapillon et al., 1995). Similarly, children under the age of 3.5 years have difficulty in forming allocentric relations among cues in order to locate a reward (Ribordy et al., 2013). The latter behavioral finding coincides with previous findings on memory and the maturation of the hippocampus in young children, such that while children under the age of 2 years are unable to form episodic memories (Bauer, 2007), children between the ages of 4 and 6 years improve dramatically in their abilities to recall the details of events (Drummey and Newcombe, 2002), possibly as a result of the hippocampal synaptic connectivity that matures at 5 years of age (Serres, 2001). Thus, it appears that the hippocampus undergoes a pivotal shift in development between 4 and 6 years of age; the very same time period in which children begin to show success on a variety of hippocampal-dependent tasks, including spatial reorientation tasks (Learmonth et al., 2008).

Although several studies have tested behavioral spatial reorientation abilities in typical individuals, it is rare to find one that used this particular spatial task with atypical participants. The most notable and recent study that has related atypical development to spatial reorientation abilities found that individuals between the ages of 9- and 27-years-old who were diagnosed with Williams Syndrome (WS), a genetic defect that affects hippocampal development and visual-spatial abilities (Bernardino et al., 2013), were impaired in their abilities to use the geometric properties of a relatively small enclosure (6.25′ × 4′) to locate a hidden toy, compared to controls (Lakusta et al., 2010). However, the participants in this study were relatively unimpaired in their abilities to use a distinct feature (i.e., colored wall) as a cue to the location of the toy. Judging by the findings of Lakusta et al. (2010), it may appear as though the hippocampus is related to the geometric processing of spaces, but not featural processing. However, in contrast with 6-year-olds in other studies on spatial reorientation (e.g., Learmonth et al., 2002), individuals with WS appear to have trouble reorienting, not only by geometry, but by features as well, indicating that an under-developed or less mature hippocampus may contribute to lower performance in using both geometric and featural information to locate objects after being disoriented.

Given that the hippocampus undergoes significant development during the first 5 years of life, then it is reasonable to suggest that an immature hippocampus would hinder a young child’s ability to reorient by using non-geometric features in the environment. This could explain why 3-year-olds have significantly more difficulty than 6-year-olds performing well on spatial reorientation tasks involving a feature. However, there is no evidence to date to directly support that this is the case. In the next section, we will briefly discuss why such data in young children is difficult to obtain. Additionally, we will discuss the advantages of using a relatively inexpensive, well-described technique known to indirectly tap into hippocampal functioning: eyeblink conditioning.

## Measuring Hippocampal Functioning in Young Children: Eyeblink Conditioning (EBC)

Although the evidence from animal and human adult studies highlights the significant role of the hippocampus in spatial navigation, and potentially spatial reorientation, it remains unclear whether developmental differences observed in spatial reorientation are mediated by developmental changes in the hippocampus. The paucity of data examining this is likely due to the difficulties and limitations of certain methodologies used to examine brain function in pediatric populations, namely EEG and *f*MRI. For example, EEG, which is a relatively inexpensive tool that has been extensively used to examine neural activation in infants and young children, does not provide the spatial resolution needed to examine subcortical regions such as the hippocampus. In contrast, *f*MRI, which provides excellent spatial resolution, has certain methodological constraints associated with it, such as having to remain still in a relatively small, enclosed space while performing a virtual task (typically). These restrictions are likely to result in participants opting out of the study due to discomfort, or failing to remain still during the task, which can render the data collected unusable. In addition, regions closest to bone-air interface, such as the hippocampus, are subject to susceptibility artifacts, which have led to functional discrepancies between *f*MRI and PET results (Devlin et al., 2000; Veltman et al., 2000). Hence, these artifacts may have played a confounding role in hippocampal activation studies with adults, suggesting that similar problems may also arise with data collected from children. Therefore, when taking the high monetary cost of *f*MRI (>$500/hour per participant) into consideration, utilizing this method to examine real-time brain function in children younger than 5 years of age is not feasible. In light of the potential difficulties, including attrition, associated with using *f*MRI, it is necessary to find alternative ways of studying children’s hippocampal development and its effect on performance on hippocampal-dependent spatial tasks. We suggest that EBC may serve as an ideal tool to examine the development of hippocampal functioning.

Classical EBC is a procedure often used to study the brain areas involved in associative learning (Cheng et al., 2008). In general, EBC occurs when a neutral stimulus (i.e., the conditioned stimulus or CS) is paired with a stimulus that elicits a biological response (i.e., the unconditioned stimulus or US) for several trials so that the subject presented with the stimuli learns the pairing between the two. As a result of this learned association, the subject will respond to the CS (i.e., tone) in relatively the same way that he or she responds to the US (i.e., puff of air to the eye), for instance, by blinking. Therefore, the overall goal of EBC is to measure whether participants can learn the temporal contingencies, or associations, between said stimuli. Two types of classical conditioning paradigms widely studied are trace EBC and delay EBC. In trace conditioning, the offset of the CS and onset of the US are separated by a silent trace period, whereas in delay conditioning, the CS and US are presented in succession, but co-terminate (Cheng et al., 2008).

One significant advantage of using EBC is that it provides a behavioral measure of brain function, with a well-defined, underlying neural circuitry that is conserved between animals and humans. Specifically, the cerebellum has been implicated in both delay and trace conditioning, while the hippocampus is primarily involved in trace conditioning (Christian and Thompson, 2003). Using animal models, it has been shown that damage to the cerebellum leads to deficits in learning acquisition during delay *and* trace conditioning (Woodruff-Pak and Disterhoft, 2008), while damage to the hippocampus results in deficits acquiring trace conditioning exclusively (Beylin et al., 2001). Consistent with these findings in animals, results from an *f*MRI study with humans showed that the cerebellum is similarly activated during both trace and delay conditioning, while the hippocampus is significantly activated during trace conditioning only (Cheng et al., 2008). Interestingly, the greatest activation was observed in the right medial temporal lobe, a region similarly important for spatial memory (Burgess et al., 2002), suggesting that similar neural mechanisms related to associative learning processes are involved in both trace EBC and spatial memory. Given that spatial reorientation relies on spatial memory, it is likely that it too shares similar underlying processes with trace EBC.

Another major advantage of employing EBC to examine hippocampal functioning is that it is relatively easy to implement in infants and young children as well as atypical pediatric populations. EBC can be successfully used with neonates (Little et al., 1984; Fifer et al., 2010; Reeb-Sutherland et al., 2011), 4–5-month-old infants (Ivkovich et al., 1999; Claflin et al., 2002), and 4–6-year-old children (Werden and Ross, 1972). In addition, EBC has been used as a tool to explore cerebellar and hippocampal functioning in several neurodevelopmental disorders (for review, see Reeb-Sutherland and Fox, 2015) in pediatric populations, including autism spectrum disorder (ASD; Sears et al., 1994; Oristaglio et al., 2013), Down syndrome (Ohlrich and Ross, 1968), fetal alcohol syndrome (FAS; Coffin et al., 2005; Jacobson et al., 2008, 2011), and attention-deficit/hyperactivity disorder (ADHD; Coffin et al., 2005; Frings et al., 2010). The fact that EBC can be employed with infants and young children as well as atypical pediatric populations, which are typically difficult to collect *f*MRI data from, demonstrates that EBC may be a reliable tool that can be implemented in children to examine the development of hippocampal functioning in relation to spatial reorientation abilities.

One criticism of using EBC to examine hippocampal functioning in relation to spatial tasks, however, is that it does not directly assess the behavior of interest (e.g., spatial reorientation) as is the case in *f*MRI studies with adults. Given the indirect nature of studying EBC in relation to hippocampal-driven behaviors, it may seem analogous to hippocampal measures obtained via structural MRI. However, in contrast to measures of hippocampal structure, EBC provides a measure of hippocampal *function* while retaining its well-defined neural circuitry and child-friendly nature. Another potential criticism of EBC is that it has been primarily used to analyze group level differences as opposed to individual differences. Recent infant studies have begun to investigate heterogeneity in associative learning via EBC (Reeb-Sutherland et al., 2012). Hence, we propose that individual differences in EBC performance should be further explored to better understand the development and role of hippocampal function in spatial task performance.

## Developmental Implications of Eyeblink Conditioning

Associative learning, or the ability to identify the causal relations between events in the environment, is crucial for adaptation and survival. These two forms of associative learning, delay and trace conditioning, have different developmental trajectories. For example, in humans, delay conditioning is present very early after birth (Little, 1973; Fifer et al., 2010; Reeb-Sutherland et al., 2011), while trace conditioning takes longer to develop, presumably due to the higher order cognitive abilities recruited during trace conditioning (Herbert et al., 2003). Newborns can learn the predictive relation between a tone and a puff of air to their eye while they sleep, suggesting that their ability to learn contingencies during sleep may be important for their cognitive development (Fifer et al., 2010). In addition, 2-month-old infants have been shown to be able to learn a delay (but not trace) conditioned response (CR) after extensive training (Little, 1973; Little et al., 1984). Standard delay conditioning, in which the delay between the onset of the CS and the onset of the US is 650 ms, is present in 5-month-old infants just as well as in adults, but long-delay (e.g., 1250 ms delay) and trace (e.g., 500 ms trace) conditioning are less robust in 5-month-olds than in adults (Herbert et al., 2003). Furthermore, typical preschool children aged 4 through 6 years have shown greater difficulty acquiring trace than delay conditioning compared to typical adults, who do not display deficits in either procedure (Werden and Ross, 1972). In addition, although performance in trace conditioning continues to improve throughout childhood, it still does not reach adult levels even by 12 years of age (Jacobson et al., 2011).

Studies demonstrating that infants acquire learning similarly to adults when they are conditioned using a delay EBC paradigm with a short delay period (i.e., 650 ms; Hoffman et al., 1985; Herbert et al., 2003) suggest that the cerebellum is sufficiently developed within the first half of the first year of life. In contrast, infants do not display adequate learning acquisition during trace conditioning until approximately 4–6 years of age (Werden and Ross, 1972), implying slower hippocampal than cerebellar development. These results further suggest that the hippocampus develops in a protracted fashion throughout childhood, consistent with the development of memory abilities-both spatial and episodic.

There are no studies to date that have systematically examined hippocampal-dependent EBC in typically developing children between 2–4 years of age, so the development of hippocampal-dependent learning and memory during this period of time still remains a mystery. Having this information would greatly inform us of whether there is indeed a shift in hippocampal development between 2–6 years of age. Furthermore, no studies to date have linked the development of hippocampal function to large-scale spatial behaviors in children, but EBC provides a novel means to do so. Hence, the use of EBC can help close a gap in our understanding of why preschool children have greater difficulty than school-aged children reorienting by a distal, non-geometric cue in order to identify the location of a hidden object.

## Conclusion and Future Directions

In the present paper, we discuss the development of spatial navigation and reorientation as well as that of EBC, and the underlying neurobiology involved in these tasks. If the hippocampus is in fact utilized during spatial reorientation tasks involving a distinct feature, not only would we be able to observe age-related differences in performance via EBC, but also predict individual trajectories in performance on certain spatial tasks. Future neuroimaging studies on children performing a virtual version of the classic reorientation task are needed in order to determine whether the hippocampus is indeed significantly less activated in preschool children than in older children during such a task. Until then it may be best to employ a simpler, indirect approach to studying hippocampal functioning, namely EBC. This technique could illuminate long-standing questions about the underlying mechanisms involved in large-scale spatial abilities in children, thus providing one explanation for the observed developmental differences in spatial reorientation tasks. More importantly, these results would broaden our understanding of early hippocampal development, primarily by closing a gap in the EBC literature, where not much data has been gathered about hippocampal-dependent EBC in typically developing children, in particular. Furthermore, we reason that the ability to detect hippocampal-dependent contingencies is likely a biomarker for success in spatial tasks that require the hippocampus, including, but not limited to, spatial reorientation. In conclusion, this developmental work can provide convergent evidence with adult and animal cognitive neuroscience, allowing us to connect rich but disparate studies on the neural and behavioral development of spatial cognition.

### Conflict of Interest Statement

The authors declare that the research was conducted in the absence of any commercial or financial relationships that could be construed as a potential conflict of interest.
